# The effect of interleukin-13 (IL-13) and interferon-γ (IFN-γ) on expression of surfactant proteins in adult human alveolar type II cells *in vitro*

**DOI:** 10.1186/1465-9921-11-157

**Published:** 2010-11-10

**Authors:** Yoko Ito, Robert J Mason

**Affiliations:** 1 Department of Medicine, National Jewish Health, 1400 Jackson Street, Denver, CO 80206, USA

## Abstract

**Background:**

Surfactant proteins are produced predominantly by alveolar type II (ATII) cells, and the expression of these proteins can be altered by cytokines and growth factors. Th1/Th2 cytokine imbalance is suggested to be important in the pathogenesis of several adult lung diseases. Recently, we developed a culture system for maintaining differentiated adult human ATII cells. Therefore, we sought to determine the effects of IL-13 and IFN-γ on the expression of surfactant proteins in adult human ATII cells *in vitro*. Additional studies were done with rat ATII cells.

**Methods:**

Adult human ATII cells were isolated from deidentified organ donors whose lungs were not suitable for transplantation and donated for medical research. The cells were cultured on a mixture of Matrigel and rat-tail collagen for 8 d with differentiation factors and human recombinant IL-13 or IFN-γ.

**Results:**

IL-13 reduced the mRNA and protein levels of surfactant protein (SP)-C, whereas IFN-γ increased the mRNA level of SP-C and proSP-C protein but not mature SP-C. Neither cytokine changed the mRNA level of SP-B but IFN-γ slightly decreased mature SP-B. IFN-γ reduced the level of the active form of cathepsin H. IL-13 also reduced the mRNA and protein levels of SP-D, whereas IFN-γ increased both mRNA and protein levels of SP-D. IL-13 did not alter SP-A, but IFN-γ slightly increased the mRNA levels of SP-A.

**Conclusions:**

We demonstrated that IL-13 and IFN-γ altered the expression of surfactant proteins in human adult ATII cells *in vitro*. IL-13 decreased SP-C and SP-D in human ATII cells, whereas IFN-γ had the opposite effect. The protein levels of mature SP-B were decreased by IFN-γ treatment, likely due to the reduction in active form cathpesin H. Similarly, the active form of cathepsin H was relatively insufficient to fully process proSP-C as IFN-γ increased the mRNA levels for SP-C and proSP-C protein, but there was no increase in mature SP-C. These observations suggest that in disease states with an overexpression of IL-13, there would be some deficiency in mature SP-C and SP-D. In disease states with an excess of IFN-γ or therapy with IFN-γ, these data suggest that there might be incomplete processing of SP-B and SP-C.

## Background

The alveolar type II (ATII) cell produces pulmonary surfactant and most of the surfactant proteins in the lung. The four surfactant proteins, SP-A, SP-B, SP-C and SP-D, have been shown to play pivotal roles in the regulation of surfactant lipid metabolism, lipid membrane organization and host defense in the lung [1]. Dysregulation of surfactant protein expression has been postulated to be important in the pathogenesis of several lung diseases [2-7]. Alterations in these proteins likely have important consequences for overall lung homeostasis and defense against pathogens.

SP-A and SP-D are water-soluble and belong to the collectin subgroup of C-type lectins [8]. SP-A genetic variants are predisposed to both interstitial pulmonary fibrosis (IPF) and lung cancer [2,3]. SP-A-/- mice show increased susceptibility to bacterial, viral and fungal pathogens but have no reported lung structural abnormalities [9]. SP-D-/- mice spontaneously develop emphysema and fibrosis, which is thought to be the result of sustained inflammation associated with abnormal oxidant metabolism and matrix metalloproteinase (MMP) activity [10]. Both SP-A and SP-D knockout mice have increased lung inflammation when they are infected with bacteria or viruses compared to wild-type strains [11]. SP-A and/or SP-D concentration in bronchoalveolar lavage fluid (BALF) are significantly decreased in patients with acute respiratory distress syndrome (ARDS), IPF, collagen vascular disease associated interstitial pneumonia, hypersensitivity pneumonia, sarcoidosis and cystic fibrosis [12-15]. Van De Graaf et al. found that SP-A is decreased in BALF from patients with bronchial asthma [16], whereas Cheng, G. et al. reported increased amounts of SP-A in both bronchial and alveolar lavage and increased levels of SP-D in alveolar lavage fluid but not bronchial lavage fluid in patients with asthma [17]. Cigarette smoking is reported to reduce SP-A and SP-D levels in BALF [18,19]. Although SP-A and SP-D are thought to be important components in innate immunity, there has not been an association of genetic deficiencies of SP-A or SP-D in humans with recurrent or persistent respiratory infections.

SP-B and SP-C are extremely hydrophobic and play critical roles in the biophysical functions of surfactant [20]. Polymorphisms of the SP-B gene are reported to be associated with squamous cell carcinoma of lung [4], risk for acute respiratory distress syndrome (ARDS) [5] and chronic obstructive pulmonary disease (COPD) [6]. Recent studies have revealed that some familial forms of pulmonary fibrosis are associated with mutations in the SP-C gene [7]. Depending on the genetic background, SP-C deficient mice can spontaneously develop chronic inflammation and have increased and prolonged pulmonary fibrosis following intratracheal instillation of bleomycin [21].

IL-13 is a pleiotropic cytokine and a major effector molecule at sites of Th2 inflammation and tissue remodeling. IL-13 is a potent stimulator of eosinophilic, lymphocytic, and macrophage-dominant inflammation, mucus metaplasia, and fibrosis. [22-27]. IL-13 dysregulation plays an important role in the pathogenesis of a variety of lung diseases including asthma, IPF, viral pneumonia, and COPD [22,28-33]. In addition, BALF from IL-13 overexpressing mice have a 3- to 6-fold increase in surfactant phospholipids, a 2- to 3-fold increase in SP-A, -B, and -C, and a 70-fold increase in SP-D [34]. In neonatal rat ATII cells, IL-4 and IL-13, but not IFN-γ, increases intracellular SP-D, but levels of other surfactant proteins were not reported [35].

IFN-γ is the prototypic Th1 cytokine and is known to play a key role in the regulation of diverse immune responses [36]. Dysregulated IFN-γ production has been implicated in a large number of diseases, which are related to inflammation and remodeling characterized by tissue atrophy and/or destruction [37]. In pulmonary emphysema, alveolar septal destruction is accompanied by increased numbers of CD8+ cells that produce IFN-γ and IFN-γ inducible protein 10/CXCL10 [38,39]. Overexpression of IFN-γ in mice causes pulmonary emphysema, which is suggested to be due to cathepsin S-dependent epithelial cell apoptosis [37,40]. In human fetal alveolar epithelial cells *in vitro*, IFN-γ is reported to increase SP-A protein levels by 3-fold and SP-A mRNA levels by 2.7-fold but does not alter SP-B and SP-C mRNA levels [41].

Although dysregulation of the Th1/Th2 cytokine is related to the pathogenesis of several adult lung diseases and alterations of surfactant proteins have been reported in variety of lung diseases, the effect of IL-13 or IFN-γ on the expression of surfactant proteins in primary adult human ATII cells has not been reported. Methods for isolating and culturing adult rat and mouse ATII cells and fetal human ATII cells have been available for years, but there has been less success in maintaining the differentiated functions of adult human alveolar epithelial cells in primary culture. A variety of methods for isolating human type II cells have been published and some of their properties have been described [42-47], but maintenance of surfactant protein expression in adult human ATII cells in monolayer culture has been difficult. Recently, we developed a system for maintaining the differentiated functions of adult human ATII cells *in vitro *[48]. Therefore, the aim of this study is to investigate the IL-13 and IFN-γ effect on the expression of the surfactant proteins using primary human adult ATII cells in monolayer culture *in vitro*. The experiments with human ATII cells were also repeated with rat ATII cells.

## Methods

### Donor information

We obtained human lungs from deidentified organ donors whose lungs were not suitable for transplantation and donated for medical research through the National Disease Research Interchange (Philadelphia, PA) and the International Institute for the Advancement of Medicine (Edison, NJ). The Committee for the Protection of Human Subjects at National Jewish Health approved this research. We selected donors with reasonable lung function with a PaO2/FIO2 ratio of >250, no history of clinical lung disease and a chest radiograph that did not indicate infection, and a limited time on the ventilator. The gender, age, and smoking history were variable and not selection criteria. The human donors used in this study included 6 males and 6 females with age ranges from 10 to 72, and there were 7 current smokers and 5 nonsmokers. Hence, there was a significant amount of variability among the donors, as expected.

### Human ATII cell isolation

We modified the human type II cell isolation method published by Fang and coworkers [44]. Briefly, the middle lobe was perfused, lavaged, and then instilled with elastase (12.9 U/ml; Roche Diagnostics, Indianapolis, IN) for 50 minutes at 37°C. The lung was minced, and the cells were isolated by filtration and partially purified by centrifugation on a discontinuous density gradient made of Optiprep (Accurate Chemical Scientific Corp., Westbury, NY) with densities of 1.080 and 1.040, and by negative selection with CD14-coated magnetic beads (Dynal Biotech ASA, Oslo, Norway) and binding to IgG-coated Petri dishes (Sigma, St. Louis, MO). The cells were counted and cytocentrifuged. Cell preparations were made to assess cell purity by staining for cytokeratin (CAM 5.2; Dako Cytomation, Carpinteria, CA). The cells were stored in 10% dimethyl sulfoxide (DMSO) and 90% fetal bovine serum (FBS) in liquid nitrogen until they were used in these studies.

### Culture of human ATII cells

The isolated cells were resuspended in Dulbecco's Modified Eagle's Medium (DMEM) supplemented with 10% FBS and 2 mM glutamine, 2.5 μg/ml amphotericin B, 100 μg/ml streptomycin, 100 units/ml penicillin G (Mediatech, Inc., Manassas, VA), and 10 μg/ml gentamicin (Sigma-Aldrich, St. Louis, MO). 4.0 million cells were plated on 4.2 cm^2 ^millicell inserts (Millipore Corp., Bedford, MA) that had been previously coated with a mixture of 50% Matrigel (BD Biosciences, Bedford, MA) and 50% rat-tail collagen in DMEM with 10% FBS [49]. For most of our studies, after 48 h the media was changed to DMEM including 5% heat inactivated human serum (Mediatech, Inc.) and 10 ng/ml TGFα (R&D Systems, Minneapolis, MN). Two days later, 10 ng/ml keratinocyte growth factor (KGF, Amgen, Thousand Oaks, CA) was added instead of TGFα for 4 d, and the medium was changed every other day. Therefore, cells in all conditions were cultured for a total of 8 d with or without human recombinant IL-13 or IFN-γ (R&D Systems) added for the last 2, 4 or 6 d. Additional studies were done with a slightly different set of differentiation factors: KGF(K), isomethylbutyl xanthene (I) and 8Br-cAMP (A) for 2 d followed by KIA and dexamethasone (D) for 4 d, designated as KIAD [48].

### Rat ATII cell isolation and culture

ATII cells were isolated from pathogen-free adult male Sprague-Dawley rats (Harlan, Indianapolis, IN) by dissociation with porcine pancreatic elastase (Roche Diagnostics) and partial purification on discontinuous density gradients by methods previously described [50]. This research was approved by the Animal Care Committee at National Jewish Health (IACUC). Type II cells were plated on 4.2 cm^2 ^millicell inserts (Millipore Corp). 2.5 million freshly isolated viable type II cells were plated in DMEM containing 5% rat serum (RS) (Pel-Freez Biologicals, Rogers, AR), 2 mM glutamine, 2.5 μg/ml amphotericin B, 100 μg/ml streptomycin, 100 units/ml penicillin G (all from Mediatech, Inc.), and 10 μg/ml gentamicin (Sigma-Aldrich). After attachment for 24 h, the cells were rinsed twice with DMEM and then cultured in DMEM containing RS, glutamine, antibiotics described above and 10 ng/ml KGF for 6 d with or without recombinant rat IL-13 (20 ng/ml) or rat IFN-γ (100 ng/ml) (R&D Systems) for the last 4 d.

### Immunoblotting and real-time PCR (RT-PCR)

Protein and mRNA expression of corresponding genes were measured by western blotting and real-time RT-PCR according to protocols as described previously [49]. Polyacrylamide gradient gels (8-16%; Invitrogen Corporation) run in tris glycine buffer were used to separate proteins. Proteins were run in the reduced state except for mature SP-B, which was run unreduced. For western blotting, protein loading was normalized to glyceraldehyde-3-phosphate dehydrogenase (GAPDH). The primary antibodies were mouse anti-human SP-A (PE-10), SP-D (1G11) (a gift from Yoshio Kuroki), rabbit anti-rat SP-A and SP-D, rabbit anti-human proSP-B, rabbit anti-sheep mature SP-B (Chemicon International, Temecula, CA), rabbit anti-human proSP-C, rabbit anti-human mature SP-C (Seven Hills Bioreagents, Cincinnati, OH), mouse anti-human ABCA3 (Seven Hills Bioreagents), mouse anti-human Cathepsin H, and mouse anti-rabbit GAPDH (abcam, Cambridge, MA). The intensities of the bands were calculated using NIH Image software (version 1.62). For real-time RT-PCR, the expression levels of genes were expressed as a ratio to the expression of the constitutive probe 36B4, acidic ribosomal phosphoprotein P0 [51]. The specific primers and probes used in these experiments are listed in Table 1.

**Table 1:** Sequence of Primer and Probes Used in This Study

Gene Name	Forward Primer	Probe	Reverse Primer
SP-A	GCCATTCAGGAGGCATGTG	CGGCCGCATTGCTGTCCCA	GCCTCATTTTCCTCTGGATTCC
SP-B	TGGGAGCCGATGACCTATG	CAAGAGTGTGAGGACATCGTCCACATCC	GCCTCCTTGGCCATCTTGT
SP-C	CGGGCAAGAAGCTGCTTCT	CCACACCGCAGGGACAAACCCT	CCACACCGCAGGGACAAACCCT
SP-D	ACACAGGCTGGTGGACAGTTG	CCTCTCCACGCTCTGCCGCGT	TGTTGCAAGGCGGCATT
36B4	CCACGCTGCTGAACATGCT	AACATCTCCCCCTTCTCCTTTGGGCTT	TCGAACACCTGCTGGATGAC

Definition of abbreviations: SP, surfactant protein

### Immunofluorescence of human proSP-C

The cells were fixed in 4% paraformaldehyde, and then the filters were embedded in paraffin as described [52]. The primary antibodies included rabbit anti-human proSP-C (Seven Hills Bioreagents). The secondary antibody was donkey Alexa Fluor 488 anti-rabbit IgG (H+L) from Invitrogen (Corporation, Carlsbad, CA).

### Statistical Analysis

All data were presented as means ± standard error of the mean. One-way ANOVA was used to compare the difference between two or more groups. Appropriate post hoc tests were selected for multiple comparison. Statistical significance was set at p < 0.05.

## Results

### Expression of surfactant proteins in adult human ATII cells cultured on Matrigel and rat tail collagen coated inserts with IL-13

**Figure 1:**
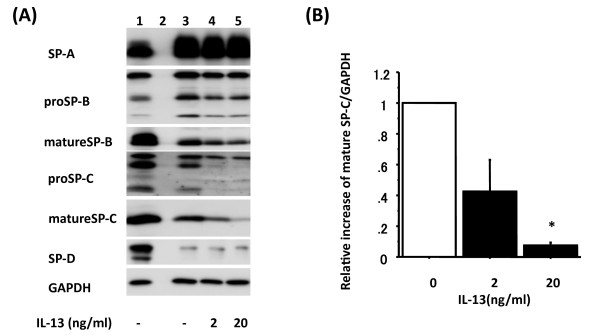
**IL-13 alters mature SP-C protein level in a dose-dependent manner**. Adult human ATII cells were cultured on Matrigel and rat-tail collagen coated inserts in DMEM containing 5% heat inactivated human serum with 2 d of TGFα followed by 4 d of KGF. Panel A shows a representative immunoblot from ATII cells cultured with 2 or 20 ng/ml IL-13 for the final 4 days. Lane 1: day 0 control (freshly isolated ATII cells), Lane 2: empty lane, Lane 3: 2 d 10 ng/ml TGFα + 4 d 10 ng/ml KGF, Lane 4: 2 d 10 ng/ml TGFα + 4 d 10 ng/ml KGF with 4 d 2 ng/ml IL-13, Lane 5: 2 d 10 ng/ml TGFα + 4 d 10 ng/ml KGF with 4 d 20 ng/ml IL-13. Panel B shows the reduction in mature SP-C protein levels after treatment with IL-13 (black) by immunoblotting data normalized to GAPDH (n = 3), which are analyzed by NIH Image. The comparison is to cultures without IL-13. Representative data are shown in Panel A lane 3 to 5. *: p < 0.05 v.s without IL-13.

**Figure 2:**
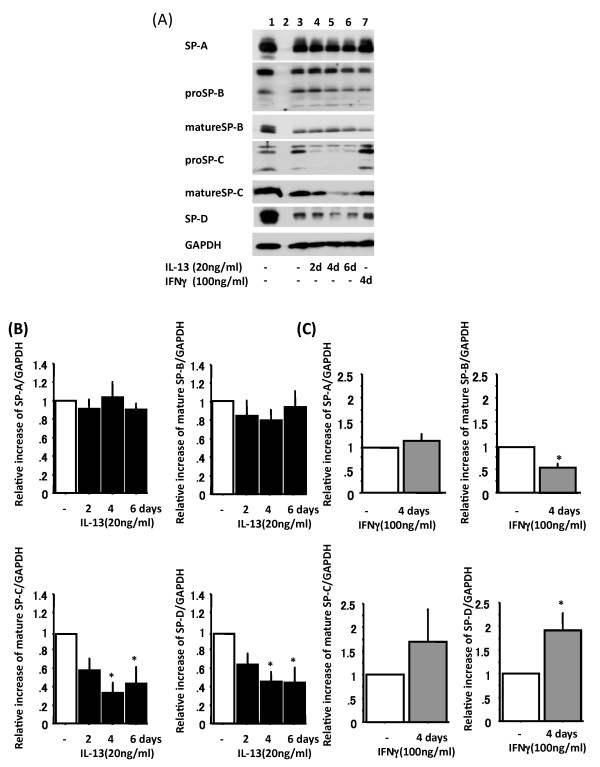
**IL-13 and IFN-γ alter surfactant protein levels**. Adult human ATII cells were cultured on Matrigel and rat-tail collagen coated inserts in DMEM containing 5% heat inactivated human serum with 2 d of TGFα followed by 4 d of KGF. Panel A shows representative immunoblot from ATII cells cultured with 20 ng/ml IL-13 for 2, 4 or 6 d or with 100 ng/ml IFN-γ for 4 d. Lane 1: day 0 control, Lane 2: empty lane, Lane 3: 2 d 10 ng/ml TGFα + 4 d 10 ng/ml KGF, Lane 4: 2 d 10 ng/ml TGFα + 4 d 10 ng/ml KGF with 2 d 20 ng/ml IL-13, Lane 5: 2 d 10 ng/ml TGFα + 4 d 10 ng/ml KGF with 4 d 20 ng/ml IL-13, Lane 6: 2 days 10 ng/ml TGFα + 4 d10 ng/ml KGF with 6 d 20 ng/ml IL-13, Lane 7: 2 days 10 ng/ml TGFα + 4 d 10 ng/ml KGF with 4 d 100 ng/ml IFN-γ. Panel B shows surfactant proteins levels from IL-13 (black) time-course treatment immunoblotting data normalized by GAPDH (n = 6), which are analyzed by NIH Image. Only SP-A, mature SP-B, mature SP-C and SP-D data are shown. The comparison is to cultures without IL-13. Representative data are shown in Panel A lane 3 to 6. *: p < 0.05 v.s without IL-13. Panel C shows surfactant proteins levels from IFNγ treatment (gray) immunoblotting data normalized by GAPDH (n = 6), which are analyzed by NIH Image. Representative data are shown in Panel A lane 3 and 7. *: p < 0.05 v.s without IFN-γ.

Human ATII cells were isolated and cultured *in vitro *for 8 d (2 d adherence, 2 d TGFα and 4 d KGF) with 2 or 20 ng/ml human recombinant IL-13. The protein level of mature SP-C showed significant dose-dependent down-regulation by human recombinant IL-13 (Figure 1A, B). Next we used 20 ng/ml human recombinant IL-13 for a time-course experiment, added it to the cultured cells for the final 2, 4 or 6 d and evaluated the expression of surfactant protein levels on day 8 by immunoblotting (n = 6) (Figure 2A, B). Protein levels of SP-A and mature SP-B were not altered by IL-13, whereas those of mature SP-C and SP-D were greatly down-regulated by 4 or 6 d of treatment with IL-13 (relative increase of mature SP-C: without IL-13 1.0, 2 d IL-13 0.60 ± 0.13, 4 d IL-13 0.35 ± 0.11p = 0.001, 6 d IL-13 0.45 ± 0.19 p = 0.005; SP-D: without IL-13 1.0, 2 d IL-13 0.66 ± 0.12, 4 d IL-13 0.47 ± 0.11 p = 0.002, 6 d IL-13 0.46 ± 0.17 p = 0.003) (Figure 2A, B).

**Figure 3:**
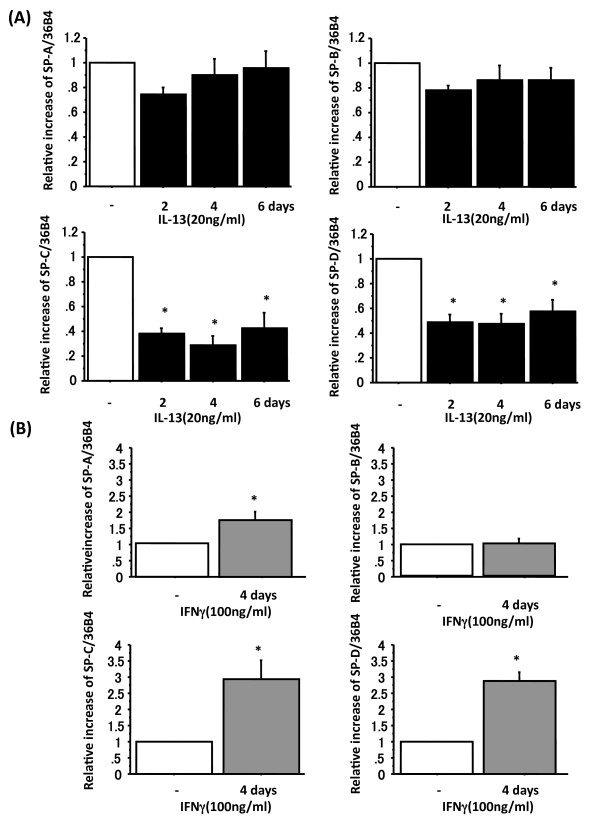
**IL-13 and IFN-γ alter surfactant protein mRNA levels**. Adult human ATII cells were cultured on Matrigel and rat-tail collagen coated inserts in DMEM containing 5% heat inactivated human serum with 2 d TGFα followed by 4 d KGF. Panel A shows mRNA data from ATII cells cultured with 20 ng/ml IL-13 (black) for 2, 4 or 6 d. mRNA levels for surfactant proteins were measured by quantitative real-time PCR and normalized to the constitutive probe 36B4 (n = 6). *: p < 0.05 v.s without IL-13. Panel B shows mRNA data from ATII cells cultured with 100 ng/ml IFN-γ (gray) for 4 d. mRNA levels for surfactant proteins were normalized to the constitutive probe 36B4 by quantitative real-time PCR (n = 6). *: p < 0.05 v.s without IFN-γ

We then assessed whether IL-13 altered the mRNA levels of surfactant proteins (n = 6) (Figure 3A). Consistent with the protein levels (Figure 2B), mRNA levels of SP-C and SP-D were significantly down-regulated in response to 2, 4 or 6 d of IL-13 treatment (relative increase of SP-C: without IL-13 1.0, 2 d IL-13 0.38 ± 0.05 p < 0.001, 4 d IL-13 0.29 ± 0.07 p < 0.001, 6 d IL-13 0.42 ± 0.13 p < 0.001; SP-D: without IL-13 1.0, 2 d IL-13 0.48 ± 0.06 p < 0.001, 4 d IL-13 0.47 ± 0.08 p < 0.001, 6 d IL-13 0.57 ± 0.10 p < 0.001) (Figure 3A). There was no change in SP-A and SP-B mRNA levels.

**Figure 4:**
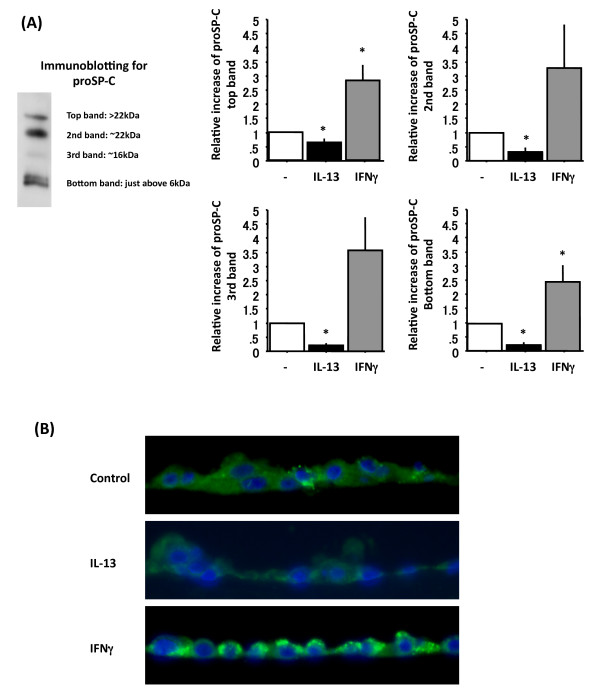
**IFN-γ increases whereas IL-13 decreases proSP-C**. Adult human ATII cells were cultured on Matrigel and rat-tail collagen coated inserts in DMEM containing 5% heat inactivated human serum for 2 d with10 ng/ml TGFα followed by 4 d 10 ng/ml KGF with or without 4 d 20 ng/ml IL-13 or 100 ng/ml IFN-γ. Panel A shows protein levels by immunoblotting for four different proSP-C bands normalized by GAPDH from 6 different donors cells stimulated by 4 d 20 ng/ml IL-13 or 100 ng/ml IFN-γ, which are analyzed by NIH Image. Representative protein levels by immunoblotting are shown on Figure 1 Panel B lane 3, 5 and 7. White bar: without IL-13 and IFN-γ, black bar: with 20 ng/ml IL-13 for 4 d, grey bar: with 100ng/ml IFN-γ for 4 d. *: p < 0.05 v.s without IL-13 or IFN-γ. Panel B shows immunocytochemistry for proSP-C. Top picture: without IL-13 or IFN-γ, middle one: with IL-13, bottom one: with IFN-γ. green: proSP-C, blue: DAPI. These three pictures were taken at the same exposure times.

We analyzed the protein level of proSP-C in the cell lysates by immunoblotting (Figure 2A) using NIH Image and examined proSP-C by immunofluorescence of the cultured cells (Figure 4A, B). The protein level of proSP-C in cell lysates from immunoblotting analysis measured by NIH Image was significantly decreased by IL-13 (relative increase of top band: 0.64 ± 0.11 p = 0.008, 2^nd ^band: 0.31 ± 0.12 p < 0.001, 3^rd ^band: 0.21 ± 0.04 p < 0.001, bottom band: 0.22 ± 0.07 p < 0.001) (Figure 4A). In proportion to the protein expression of mature SP-C, the immunofluorescent level of proSP-C in the cells treated with IL-13 was markedly lower than cultures without IL-13 (Figure 4B top and middle pictures).

### Expression of surfactant proteins in adult human alveolar type II cells cultured on Matrigel and rat-tail collagen coated inserts with IFN-γ

Human ATII cells were isolated and cultured *in vitro *for 8 d (2 d adherence, 2 d TGFα and 4 d KGF) with 100 ng/ml human recombinant IFN-γ for the last 4 d (n = 6). We used 4 d of 100 ng/ml IFN-γ for this experiment based on our previous experiments with the KIAD system (Additional File 1A right panel). The protein levels of surfactant proteins in the cell lysates were measured by immunoblotting (Figure 2A lane 3 and 7 and Figure 2C). SP-A and mature SP-C protein levels were not changed by IFN-γ, whereas mature SP-B protein level was down-regulated (relative increase of mature SP-B: without IFN-γ 1.0, with IFN-γ 0.55 ± 0.09 p < 0.001) and SP-D protein levels were significantly up-regulated (relative increase of SP-D: without IFN-γ 1.0, with IFN-γ 1.92 ± 0.36 p = 0.028) (Figure 2A lane 3 and 7, Figure 2C). We then assessed the IFN-γ effect on mRNA levels of surfactant proteins (n = 6). The mRNA levels of SP-A, -C and -D were increased by IFN-γ (relative increase of SP-A: without IFN-γ 1.0, with IFN-γ 1.68 ± 0.26 p = 0.024, SP-C: 2.93 ± 0.60 p = 0.009, SP-D: 2.87 ± 0.28 p < 0.001) in response to 4 d of IFN-γ stimulation (Figure 3B). There was no change in SP-B mRNA level.

We also analyzed protein level of proSP-C in the cell lysates by immunoblotting (Figure 2A) using NIH Image and examined proSP-C by immunofluorescence of the cultured cells (Figure 4A, B). The protein level of proSP-C in cell lysate from immunoblotting analysed by NIH Image was also greatly increased by IFN-γ treatment (relative increase of top band: 2.86 ± 0.52 p = 0.005, 2^nd ^band: 3.28 ± 1.50 p > 0.05, 3^rd ^band: 3.58 ± 1.1 p > 0.05, bottom band: 2.53 ± 0.59 p = 0.040) (Figure 4A). The immunofluorescent level of proSP-C in the cells cultured with IFN-γ was remarkably higher than in cells without IFN-γ (Figure 4B top and bottom pictures), and IFN-γ treated cells possessed highly stained small dots in the cells, presumably membranous vesicles.

### Expression of cathepsin H and ATP binding cassette transporter A3 (ABCA3) in adult human ATII cells cultured with IL-13 or IFN-γ

**Figure 5:**
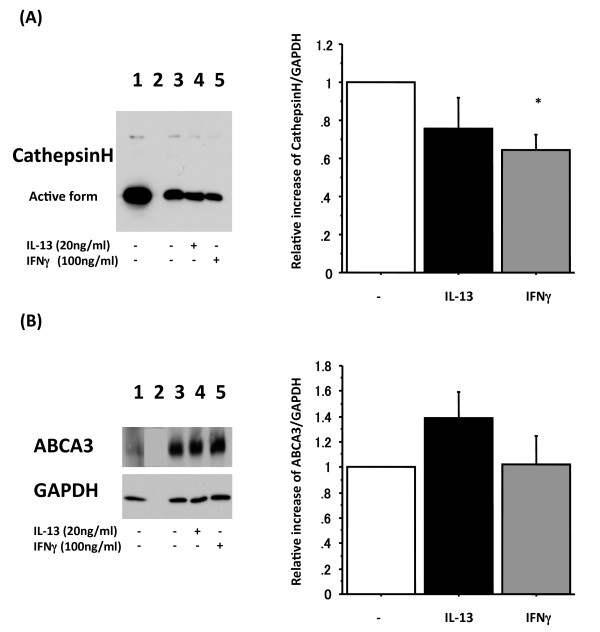
**IFN-γ reduces cathepsin H but not ABCA3**. Adult human ATII cells cultured on Matrigel and rat-tail collagen in DMEM containing 5% heat inactivated human serum with 2 d TGFα followed by 4 d KGF with 20 ng/ml IL-13 or with 100 ng/ml IFN-γ for the final 4 days. Panel A shows representative active form of cathepsin H protein levels by immunoblotting and protein levels by immunoblotting for the active form cathepsin H normalized by GAPDH from 6 different donors, which are analyzed by NIH Image. Lane 1: day 0 control, Lane 2: empty, Lane 3: 2 d 10 ng/ml TGFα + 4 d 10 ng/ml KGF, Lane 4: 2 d 10 ng/ml TGFα + 4 d 10 ng/ml KGF with 4 d 20 ng/ml IL-13, Lane 5: 2 d 10 ng/ml TGFα + 4 d 10 ng/ml KGF with 4 d 100 ng/ml IFN-γ. White bar: without IL-13 and IFN-γ, black bar: with 20 ng/ml IL-13 for 4 d, gray bar: with 100 ng/ml IFN-γ for 4 d. *: p < 0.05 v.s. without IL-13 and IFN-γ. Panel B shows representative ABCA3 protein levels by immunoblotting and protein levels by immunoblotting for ABCA3 normalized by GAPDH from 6 different donors, which are analyzed by NIH Image. Lane order is same as in Panel A. White bar: without IL-13 and IFN-γ, black bar: with 20 ng/ml IL-13 for 4 d, gray bar: with 100 ng/ml IFN-γ for 4 d. *: p < 0.05 v.s. without IL-13 and IFN-γ.

SP-B and SP-C are synthesized by ATII cell as proSP-B and proSP-C, which are proteolytically processed to mature SP-B and SP-C on route from its site of synthesis to the lamellar bodies [53,54]. Cathepsin H is one of the cysteine proteases involved in the processing of proSP-B and proSP-C. The first N-terminal processing step of proSP-C occurs in the electron-dense multivesicular bodies of ATII cells [53,54]. Because IFN-γ decreased protein levels of mature SP-B without a change in SP-B mRNA, and significantly up-regulated SP-C mRNA and proSP-C protein without an increase in mature SP-C (Figure 2C, 3B and 4A), we examined whether IFN-γ changed the level of cathepsin H in ATII cells. The active form has a molecular size of 28 kDa [55], and this band was reduced by IFNγ but not by IL-13 (Figure 5A). However, there is no change in pepsinogen C or napsin A, other proteases involved in the processing of proSP-C (data not shown).

ABCA3 is predominantly expressed in ATII cells and has been localized to the limiting membrane of lamellar bodies, which are the main intracellular storage organelle for pulmonary surfactant [56]. The protein expression of ABCA3 was measured by immunoblotting, and it was not altered by either IL-13 or IFN-γ (Figure 5B), which suggests the effect of IL-13 and IFN-γ primarily alter surfactant B and C processing and not lamellar body formation per se.

### Verification of these findings with other culture conditions in human ATII cells and results with rat ATII cells

The results above were all done with adult human ATII cells cultured with 2 d of TGFα and 4 d of KGF. Similar findings were observed in ATII cells cultured in KGF (K) alone, in 2 d of K and 2 d of KD (data not shown) or in 2 d of KIA and 4 d of KIAD (Additional File 1A and 1B). The major difference was that in the untreated conditions, the 2 d of TGFα followed by 4 d of KGF showed slightly higher levels of mature SP-C, so that it was easier to demonstrate a decrease with IL-13. Additionally, the expression of mature SP-B was not altered in the KIA + KIAD system by IL-13 or IFN-γ (Additional File 1A, B, Additional File 2). In all conditions tested, IL-13 reduced the levels of proSP-C and mature SP-C and IFN-γ increased the level of proSP-C. Hence, the effects of IL-13 and IFNγ on SP-C did not depend on the differentiation factors that were used for the human ATII cells.

We cultured rat ATII for 6 d of KGF in DMEM including 5% RS with the last 4 d recombinant IL-13 or IFN-γ. The results from rat ATII cells were slightly different from the observations with human ATII cells. In rat ATII cells, IL-13 reduced expression of SP-A, mature SP-B, proSP-C and mature SP-C but not SP-D (Additional File 3A, B, Additional File 2). IFN-γ increased SP-A, proSP-C and SP-D but not mature SP-C (Additional File 3A, B, Additional File 2). There was clearly incomplete processing of proSP-C, as indicated by the abundance of the ~22 kDa intermediate. The effects on proSP-C and mature SP-C by IL-13 and IFN-γ were similar in rat and human ATII cells, whereas the effects on other surfactant proteins were not (Additional File 2).

## Discussion

In this study, we showed that Th1/Th2 cytokines individually modulate the expression of surfactant proteins in adult human ATII cells. IL-13 reduced both mRNA and protein levels of SP-C and SP-D but did not alter those of SP-A and SP-B. Interestingly, IFN-γ up-regulated the mRNA level of SP-C and protein level of proSP-C without an increase in mature SP-C. This indicates an alteration in the processing of proSP-C. These changes were accompanied by down-regulation of the active form of cathepsin H, which is thought to be required for the processing of both proSP-B and proSP-C.

We used a different culture system for human type II cells from the one used in our previous report [48] and added TGFα to the culture system in this study. Maintenance of expression of both pro and mature SP-C is difficult with adult human ATII cells. Since SP-C levels were most significantly modified by IL-13 and IFN-γ in this study, we tried several different combinations of additives including the KIAD system to increase the expression of mature SP-C. The changes in proSP-C by IL-13 and IFN-γ were similar in all culture systems, but the expression of mature SP-C was low in the KIAD system. Therefore, we chose the culture system using 2 d 10 ng/ml TGF-α followed by 4 d 10 ng/ml KGF in DMEM with 5% heat inactivated human serum, because this system was the best system for maintaining expression of mature SP-C (Figure 1A Lane 3 and 2A Lane 3).

The mechanism whereby IL-13 or IFN-γ alters the expression of surfactant protein expression is not completely defined. However, alterations in surfactant protein levels can occur as a change in production, catabolism, processing (in terms of SP-B and SP-C) or a combination of several abnormalities. In this study, IL-13 reduced both mRNA and protein levels of SP-C and SP-D in human adult ATII cells. Our results are different from previous reports in rodents, which show that IL-13-overexpression in mice increases SP-A, SP-B, SP-C and SP-D [34] and that 4 d of 20 ng/ml IL-13 increases the intracellular SP-D protein level when compared to untreated cells in rat neonatal ATII cells *in vitro *[35]. We also performed similar experiments *in vitro *with rat adult ATII cells and found that rat IL-13 reduced the protein levels of SP-A, mature SP-B, proSP-C and mature SP-C, and did not change that of SP-D (Additional File 3). Although the differences in the expression of surfactant proteins in response to IL-13 between with human and rat cells *in vitro *is most likely due to the species differences, it might also be due to the age of animals or the experimental systems. The differences among overexpressing mice, the human studies *in vitro *and rat studies *in vitro *is complicated because of the duration of exposure, the dose, compensatory mechanisms, and systemic effects as well as other confounding factors.

IL-13 has been known to play a pivotal role in the pathogenesis of lung disease such as asthma, IPF, viral pneumonia and COPD [22,28-33]. SP-D is an important component of innate immunity in the lungs [1]. Although it is difficult to explain the pathogenesis of lung diseases due to the dysregulation of only one cytokine, the down-regulation of SP-D in response to IL-13 might modify the pathogenesis of various diseases and may also alter the susceptibility to pathogens in patients with these diseases. IPF is proposed to result from multiple cycles of alveolar epithelial cell injury and activation instead of chronic inflammatory alveolitis [57]. IL-13 is found at elevated levels in the alveolar macrophages of IPF patients [30]. It has also been showed that human fibroblasts from patients with IPF are hyper-responsive to IL-13 [58]. Additionally, a high level of IL-13Rα2 expression is detected in the lung epithelium, interstitium, and in mononuclear cells in surgical lung biopsies from patients with IPF [59]. IL-13 is elevated after administration of bleomycin in murine lungs and enhanced IL-13Rα2 signaling is thought to be involved in bleomycin-induced lung fibrosis [60]. Moreover, some familial forms of pulmonary fibrosis are associated with mutations in the SP-C gene [7]. Increased and prolonged pulmonary fibrosis following intratracheal bleomycin injection is detected in SP-C -/- mice [21]. Our data reveal that SP-C expression in adult human ATII cells is reduced in response to IL-13. Taken together, it is possible that macrophages from patients with IPF produce IL-13, which decreases the expression of SP-C in ATII cells and initiates the development of IPF by producing alveolar instability, because SP-C helps lower the surface tension in the alveolar space. Importantly, anti-IL-13 therapeutic approaches to IPF (QAX576, Novartis, phase II clinical trial) have completed, and we await the results.

SP-B and SP-C are synthesized by ATII cells as proproteins (proSP-B and proSP-C) that are processed to mature SP-B and SP-C by serial cleavage of NH_2_- and COOH- terminal peptides [53,54,61-63]. The first protease to be associated with proSP-B processing was cathepsin H [53,61] and inhibition of cathepsin H results in decreased production of mature SP-B. Napsin A and pepsinogen C, asparatic proteases, are also involved in the processing of proSP-B [53,63]. We found no changes in the protein levels of napsin A or pepsinogen C with IL-13 or IFN-γ in our studies (data not shown). On the other hand, the proteolytic processing of proSP-C to mature SP-C requires at least two distinct cleavages of the C-terminal propeptide followed by at least two cleavages of the N-terminal propeptide [64,65]. In the human lung, cathepsin H is involved in the first N-terminal processing steps of proSP-C in the electron dense multivesicular bodies in ATII cells [54], but the proteases for cleavage of other sites are still unknown. Therefore, considering proSP-B processing steps, we postulate that the protein levels of mature SP-B are slightly reduced by IFN-γ treatment in the TGFα + KGF system due to the reduction of the active form cathepesin H. Similarly, the reduction in cathepsin H, along with the increase in SP-C mRNA, produces a significant accumulation of proSP-C without an increase in mature SP-C. Zheng, T., et al. reported that IFN-γ causes protease-antiprotease abnormalities [40], alveolar epithelial cell DNA injury and apoptosis via a cathepsin S-dependent pathway that leads to emphysema in the murine lung [37]. In the latter paper, IFN-γ overexpression in mice resulted in an increase in cathepsin H mRNA levels, which appear inconsistent with our results. However, their experiment used mice, sustained overexpression of IL-13, and analyzed cathepsin H mRNA levels in the whole lung. Therefore there are many differences between their experiments and ours. Similarly, in terms of the expression of surfactant proteins in response to IFN-γ, our results differ from a previous report that showed in human fetal alveolar epithelial cells, IFN-γ increased the SP-A protein level and mRNA levels, but did not affect SP-B and SP-C mRNA levels [41]. However, again there are many differences in experimental design that could account for the different results.

IFN-γ production is stimulated by the innate immune response during infection, which alters macrophage function including enhanced pathogen recognition, antigen processing and presentation, stimulation of leukocyte recruitment, and activation of microbicidal effector functions and an anti-viral state [36]. In this study, we demonstrated that IFN-γ induces SP-D expression in adult human ATII cells. Likewise, the dual function NADPH oxides/heme peroxidase (DUOX) 2 which is an inducible DUOX form in the human primary tracheobronchial epithelial cells and important for monitoring pathologic changes in the respiratory tract, is highly up-regulated by IFN-γ, poly (I:C), or rhinovirus [66]. Considered together, it is suggested that IFN-γ promotes the host defense mechanism not only in primary immune cells but also in respiratory epithelial cells.

ABCA3 is necessary for lamellar body biogenesis, SP-B processing, and lung development late in gestation [67]. ABCA3 deficiency in human and mice leads to decreased phosphatidylcholine and phosphatidylglycerol in surfactant, dysgenesis of lamellar bodies, and respiratory distress [56,68]. IL-13 and IFN-γ alter SP-B, SP-C and cathepsin H expression diversely in our study. We thus hypothesized that these cytokines would alter lamellar body function. However, as shown in Figure 5B, ABCA3 expression was not changed by these cytokines. These results focus the effect of IL-13 and IFN-γ on processing of SP-B and SP-C and not lamellar body genesis per se.

The results with rat cells were differed from the observations with human cells in several ways (Additional File 2). Therefore, it is important to confirm observations made with rodent cells with adult human cells, if the results with rodent cells are to be used to explain the pathogenesis of adult human lung diseases.

## Conclusions

We demonstrate that IL-13 and IFN-γ alter the expression of surfactant proteins in human adult ATII cells *in vitro*. Our current study suggests that these cytokines imbalance, that is Th1/Th2 cytokine imbalance, might contribute to the pathogenesis of lung diseases by disordering homeostasis in the alveolar space.

## Competing interests

The authors declare that they have no competing interests.

## Authors' contributions

YI carried out all human and rat ATII cells studies. YI and RJM participated in the design of the study and data analysis. All authors have read and approved of the final manuscript.

## Supplementary Material

Additional file 1 **Effect of IL-13 and IFN-γ on expression of surfactant proteins in adult human ATII cells cultured with a differentiation factors**. Click here for file

Additional File 2 **Summary of the result from immunoblot data**. Click here for file

Additional File 3 **IL-13 and IFN-γ alter surfactant proteins expression in adult rat ATII cells**. Click here for file
