# Supplementary material for: The effect of interleukin-13 (IL-13) and interferon-γ (IFN-γ) on expression of surfactant proteins in adult human alveolar type II cells in vitro
Source: Respir Res. 2010 Nov 10;11(1):157. doi: 10.1186/1465-9921-11-157 (PMC2992502; doi:10.1186/1465-9921-11-157)
Supplement: Additional file 1 — Effect of IL-13 and IFN-γ on expression of surfactant proteins in adult human ATII cells cultured with a differentiation factors. [file 1465-9921-11-157-S1.PDF]

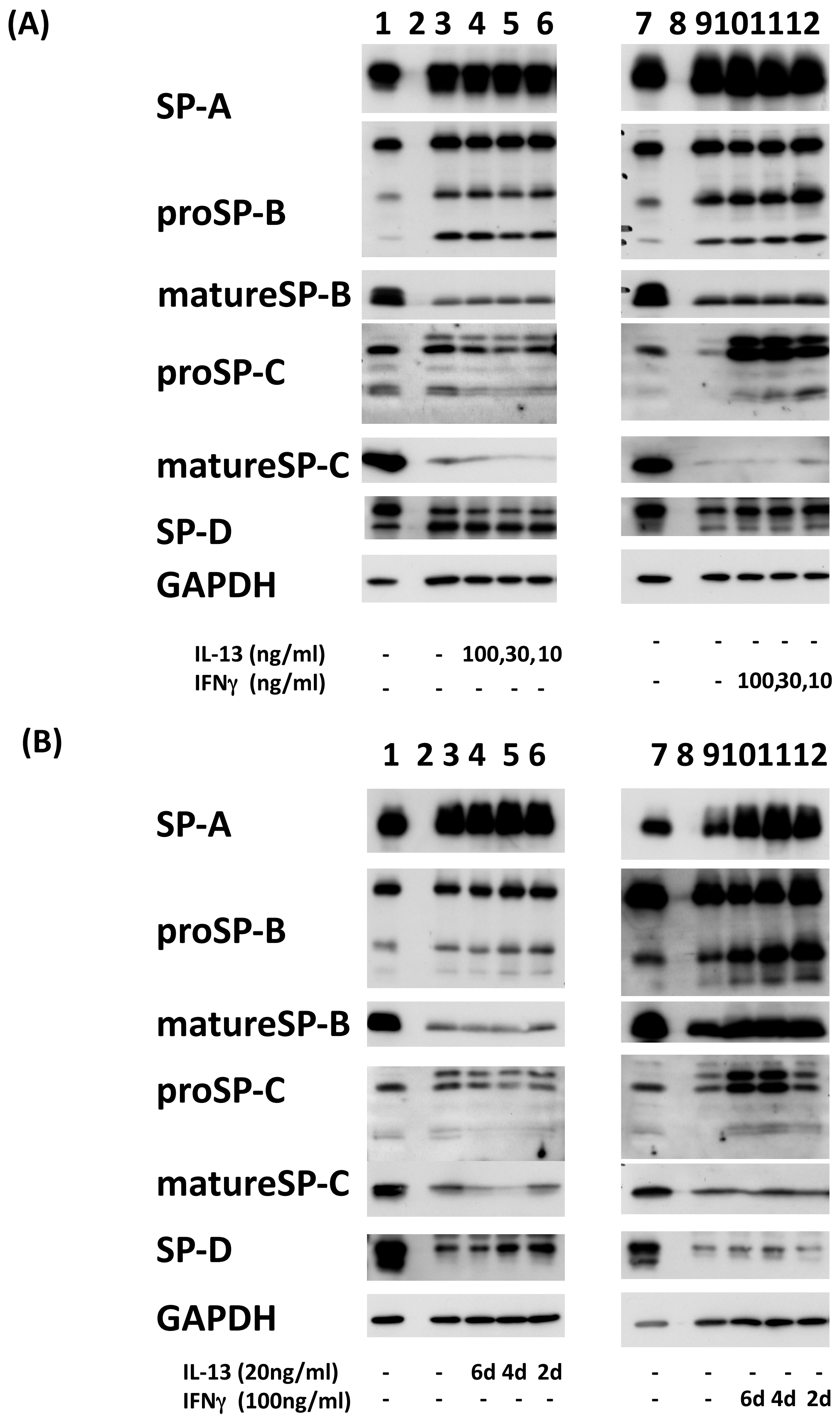
(A)
1 2 3 4 5 6
7 8 9101112
SP-A
proSP-B
matureSP-B
proSP-C
matureSP-C
SP-D
GAPDH
IL-13 (ng/ml) - - 100,30,10
IFNγ (ng/ml) - - - - -
- - - - -
- - 100,30,10
(B)
1 2 3 4 5 6
7 8 9101112
SP-A
proSP-B
matureSP-B
proSP-C
matureSP-C
SP-D
GAPDH
IL-13 (20ng/ml) - - 6d 4d 2d
IFNγ (100ng/ml) - - - - -
- - - - -
- - 6d 4d 2d

Additional File 1. Effect of IL-13 and IFN-γ on expression of surfactant proteins in adult human ATII cells cultured with a different set of differentiation factors.

Adult human ATII cells were cultured on Matrigel and rat-tail collagen coated inserts in DMEM containing 5% heat inactivated human serum with 2 d KIA followed by 4 d KIAD [48]. Panel A shows a representative immunoblot from ATII cells cultured with 10, 30 or 100 ng/ml IL-13 or IFNγ for 4 d (dose response). Lane 1: day 0 control (freshly isolated ATII cells), Lane 2: empty lane, Lane 3: 2 d KIA + 4 d KIAD, Lane 4: 2 d KIA + 4 d KIAD with 4 d 100 ng/ml IL-13, Lane 5: 2 d KIA + 4 d KIAD with 4 d 30 ng/ml IL-13, Lane 6: 2 d KIA + 4 d KIAD with 4 d 10 ng/ml IL-13 Lane 7: day 0 control (freshly isolated ATII cells), Lane 8: empty lane, Lane 9: 2 d KIA + 4 d KIAD, Lane 10: 2 d KIA + 4 d KIAD with 4 d 100 ng/ml IFN-γ, Lane 11: 2 d KIA + 4 d KIAD with 4 d 30 ng/ml IFN-γ, Lane 12: 2 d KIA + 4 d KIAD with 4 d 10 ng/ml IFN-γ. Panel B shows a representative immunoblot from ATII cells cultured with 10 ng/ml IL-13 or 100 ng/ml IFN-γ for 2, 4 or 6 d (time course). Lane 1: day 0 control (freshly isolated ATII cells), Lane 2: empty lane, Lane 3: 2 d KIA + 4 d KIAD, Lane 4: 2 d KIA + 4 d KIAD with 6 d 10 ng/ml IL-13, Lane 5: 2 d KIA + 4 d KIAD with 4 d 10 ng/ml IL-13, Lane 6: 2 d KIA + 4 d KIAD with 2 d 10 ng/ml IL-13 Lane 7: day 0 control (freshly isolated type II cells), Lane 8: empty lane, Lane 9: 2 d KIA + 4 d KIAD, Lane 10: 2 d KIA + 4 d KIAD with 6 d 100 ng/ml IFN-γ, Lane 11: 2 d KIA + 4 d KIAD with 4 d 100 ng/ml IFN-γ, Lane 12: 2 d KIA + 4 d KIAD with 2 d 100 ng/ml IFN-γ.
