# Supplementary material for: The effect of interleukin-13 (IL-13) and interferon-γ (IFN-γ) on expression of surfactant proteins in adult human alveolar type II cells in vitro
Source: Respir Res. 2010 Nov 10;11(1):157. doi: 10.1186/1465-9921-11-157 (PMC2992502; doi:10.1186/1465-9921-11-157)
Supplement: Additional File 2 — Summary of the result from immunoblot data. [file 1465-9921-11-157-S2.PDF]

Additional File 2.

Summary of the result from immunoblot data

	IL-13			IFN-γ		
**Cell types**	Human ATII cells	Human ATII cells	Rat ATII cells	Human ATII cells	Human ATII cells	Rat ATII cells
**Additives**	TGFα + K	KIA + KIAD	K	TGFα + K	KIA + KIAD	K
**SP-A**	-	-	↓	-	-	↑
**proSP-B**	-	-	NA	-	-	NA
**mature SP-B**	-	-	↓	↓	-	-
**proSP-C**	↓	↓	↓	↑	↑	↑
**mature SP-C**	↓	↓	↓	-	-	-
**SP-D**	↓	↓	-	↑	↑	↑
