# Supplementary material for: The effect of interleukin-13 (IL-13) and interferon-γ (IFN-γ) on expression of surfactant proteins in adult human alveolar type II cells in vitro
Source: Respir Res. 2010 Nov 10;11(1):157. doi: 10.1186/1465-9921-11-157 (PMC2992502; doi:10.1186/1465-9921-11-157)
Supplement: Additional File 3 — IL-13 and IFN-γ alter surfactant proteins expression in adult rat ATII cells. [file 1465-9921-11-157-S3.PDF]

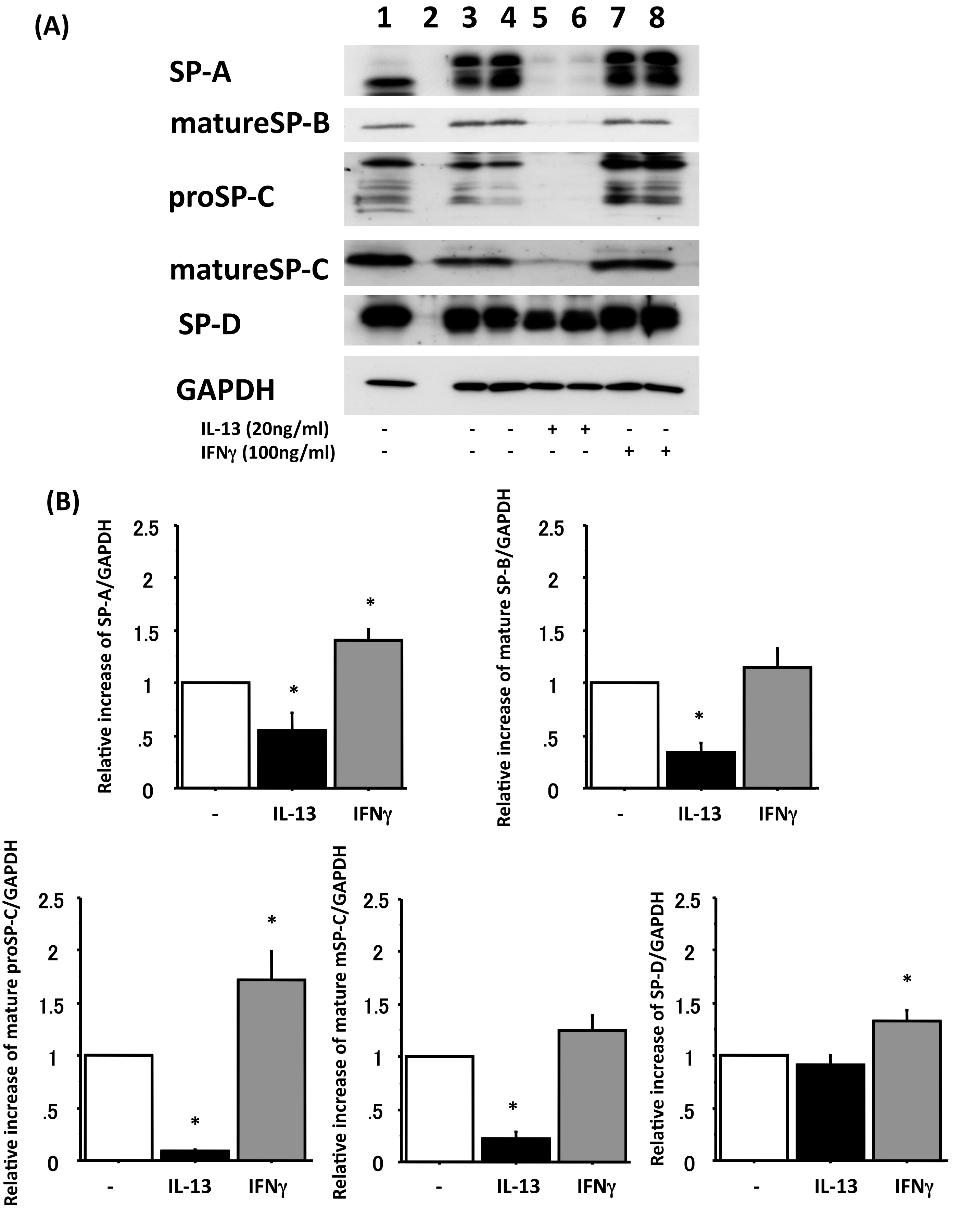
(A)
1 2 3 4 5 6 7 8
SP-A
matureSP-B
proSP-C
matureSP-C
SP-D
GAPDH
IL-13 (20ng/ml) - - - + + - -
IFNγ (100ng/ml) - - - - - + +
(B)
Relative increase of SP-A/GAPDH
Relative increase of mature SP-B/GAPDH
Relative increase of mature proSP-C/GAPDH
Relative increase of mature mSP-C/GAPDH
Relative increase of SP-D/GAPDH
2.5
2
1.5
1
.5
0
*
-
IL-13
IFNγ

Additional File 3. IL-13 and IFN-γ alter surfactant protein expression in adult rat ATII cells.

Panel A shows representative immunoblot from adult rat ATII cells cultured on Matrigel and rat-tail collagen coated inserts in DMEM containing 5% RS and 10 ng/ml KGF(K) with 20 ng/ml IL-13 (black) for 4 d or with 100 ng/ml IFN-γ (gray) for 4 d. Lane 1:day 0 control (freshly isolated ATII cells), Lane 2: empty lane, Lane 3-4: 6 d 10ng/ml KGF, Lane 5-6: 6 d 10ng/ml KGF with 4 d 20ng/ml IL-13, Lane 7-8: 6 d 10ng/ml KGF with 4 d 100ng/ml IFNγ. Panel B shows surfactant protein levels in adult rat ATII cells from immunoblotting data normalized by GAPDH (n=3), which are analyzed by NIH Image. Representative data are shown in Panel A. White bar: without IL-13 and IFN-γ, black bar: with 20 ng/ml IL-13 for 4 d, grey bar: with 100ng/ml IFN-γ for 4 d. *: $p<0.05$ v.s without IL-13 and IFN-γ.
